# Feasibility and Cost Analysis of Ambulatory Endovascular Aneurysm Repair

**DOI:** 10.1177/15266028221133694

**Published:** 2022-11-08

**Authors:** Ahmed A. Naiem, R.J. Doonan, Andre Guigui, Daniel I. Obrand, Jason P. Bayne, Kent S. MacKenzie, Oren K. Steinmetz, Elie Girsowicz, Heather L. Gill

**Affiliations:** 1 Division of vascular surgery, Royal Victoria Hospital, McGill University, Montreal, QC, Canada; 2 Division of vascular surgery, Royal Victoria Hospital, McGill University Health Centre, Montreal, QC, Canada; 3 Financial systems and process improvement finance, McGill University Health Centre, Montreal, QC, Canada; 4 Division of vascular surgery, Jewish General Hospital, Montreal, QC, Canada

**Keywords:** endovascular aneurysm repair, hospital costs, same-day discharge, cost analysis, abdominal aortic aneurysm

## Abstract

**Purpose:**

We sought to compare the costs of ambulatory endovascular aneurysm repair (a-EVAR) and inpatient EVAR (i-EVAR) at up to 1-year of follow-up.

**Materials and Methods:**

A retrospective cohort study of consecutive patients undergoing elective EVAR between April 2016 and December 2018 at two academic centers. Patients planned for a-EVAR were compared with i-EVAR. Costs at 30 days and 1 year were extracted. These included operating room (OR) use, bed occupancy, laboratory and imaging, emergency department (ED) visits, readmissions, and reinterventions. Baseline characteristics were compared. Multiple regression model was used to identify predictors of increased EVAR costs. Repeated measures analysis of variance (ANOVA) was used to compare cost differences at 30 days and 1 year via an intention-to-treat analysis. Bonferroni post hoc test compared between-group differences. A p value<0.05 was considered statistically significant.

**Results:**

One hundred seventy patients were included. Most underwent percutaneous EVAR (>94%) under spinal anesthesia (>84%). Ambulatory endovascular aneurysm repair was successful in 84% (84/100). Ambulatory endovascular aneurysm repair patients (76±8 years) were younger than i-EVAR (78±9 years). They also had a smaller mean aneurysm diameter (56±6 mm) compared with i-EVAR (59±6 mm). Emergency department visits, readmissions, and reinterventions were similar up to 1 year (all p=NS). Ambulatory endovascular aneurysm repair costs showed a non-statistically significant reduction in total costs at 30 days and 1 year by 27% and 21%, respectively. Patients younger than 85 years and males had a 30-day cost reduction by 34% (p=0.027) and 33% (p=0.035), respectively with a-EVAR.

**Conclusions:**

Same-day discharge is feasible and successful in selected patients. Patients younger than 85 years and males have a short-term cost benefit with EVAR done in the ambulatory setting without increased complications or reinterventions.

**Clinical Impact:**

This study shows the overall safety of ambulatory EVAR with proper patient selection. These patient had similar post-intervention complications to inpatients. Same day discharge also resulted in short-term reduction in costs in male patients and patients younger than 85 years.

## Introduction

Endovascular aneurysm repair (EVAR) has been a rapidly adopted technology with lower morbidity and mortality compared with open surgical repair (OSR) of abdominal aortic aneurysms (AAAs) in both randomized and non-randomized studies.^1–4^ Endovascular aneurysm repair also offers the advantage of a shortened hospital length of stay (LOS) and has surpassed OSR in terms of procedure volume.^1,5^ The value gained from lower morbidity, mortality, and LOS is met with high endografts’ costs, imaging surveillance costs, and reintervention rates.^6–8^ Data from the Open Versus Endovascular Repair (OVER) trial suggests that EVAR is still less expensive than OSR at 2 years.^9^ On the other hand, Endovascular Aneurysm Repair Trial 1 (EVAR-1) suggests that it is costlier than OSR beyond 10 years with hospital stay amongst drivers of cost.^1^ Despite these mixed results, there is an observed reduction in EVAR costs over time.^10^ Bed occupancy constitutes up to 20% of total hospital costs in EVAR even with shorter LOS.^2,11–13^ Hence, shortening LOS emerged as a strategy to reduce costs.^14,15^ Our aim was to determine whether ambulatory (a-EVAR) will cost less than inpatient (i-EVAR) at 30 days and 1 year post-EVAR.

## Materials and Methods

Consecutive patients scheduled for elective EVAR at two academic centers between April 2016 and December 2018 were retrospectively analyzed. Institutional ethical approvals were obtained. The inclusion criteria, based on our previous published experience studying the clinical safety of ambulatory EVAR,^16^ was: infrarenal AAA planned for elective EVAR, availability of a caregiver for the first 24 hours after discharge who is reliable to call or return the patient to hospital in the event of a complication. Thoraco-abdominal aortic repairs, thoracic aortic repair, and non-elective EVAR were excluded.

### Data Collection

Average costs encompassed operating room (OR), hospital stay, emergency department (ED) visits, reinterventions, and readmissions. The perioperative period was defined as the time between index visit up to 30 days. Variables were retrospectively collected, including demographics, perioperative costs, and 1-year costs. All costs are reported in Canadian dollars (1 Canadian dollar equals to 0.79 US dollars and 0.70 Euros).

### Data Analysis

Analysis was performed in an intention-to-treat manner comparing a-EVAR versus i-EVAR. Baseline characteristics were compared using independent *t*-test, chi-square test, and Mann-Whitney *U* test when appropriate. Continuous variables were reported as mean with standard deviation or median with inter-quartile range depending on data distribution. Multiple regression model was used to identify predictors of increased EVAR costs. Repeated measures analysis of variance (ANOVA) was used to compare cost differences at 30 days and 1 year via an intention-to-treat analysis. Bonferroni post hoc test compared between-group differences. Analysis was performed using Statistical Package for the Social Sciences (SPSS) Version 27 (IBM, Armonk, NY, USA). A p value<0.05 was considered statistically significant.

## Results

### Baseline Characteristics

The baseline and procedural characteristics are shown in Table 1. Ambulatory endovascular aneurysm repair accounted for 59% (n=100/170) of the patients. Patients in the a-EVAR cohort had a smaller AAA (56±6 vs 59±6 mm, p=0.011) and shorter median LOS (0.5 vs 1.3 days, p<0.001).

**Table 1.** Baseline Characteristics of a-EVAR and i-EVAR Patients.

	a-EVAR (n=100)	i-EVAR (n=70)	p value
Age in years, mean (SD)	76 ^(8)^	78 ^(9)^	0.101
Male sex, n (%)	81 (81%)	57 (81%)	0.944
Comorbidities, n(%)			
DM	18 (18%)	18 (26%)	0.226
Hypertension	72 (72%)	53 (76%)	0.589
Dyslipidemia	45 (45%)	37 (53%)	0.313
CAD	34 (34%)	23 (33%)	0.877
CKD	14 (14%)	13 (19%)	0.422
Stroke	4 (4%)	8 (11%)	0.063
Cancer	16 (16%)	12 (17%)	0.843
COPD	25 (25%)	23 (33%)	0.263
CHF	4 (4%)	7 (10%)	0.118
Arrhythmia	16 (16%)	13 (19%)	0.661
AAA diameter in mm, mean (SD)	56 (6)	59 (6)	0.011
LOS in days, median (IQR)	0.5 (0.4–0.8)	1.3 (1.2–2.3)	<0.001
Anesthesia type, n (%)			0.950
General	10 (10%)	8 (11%)	
Spinal	86 (86%)	59 (84%)	
Local	4 (4%)	3 (4%)	
Percutaneous access, n (%)	96 (96%)	66 (94%)	0.603

Abbreviations: AAA, abdominal aortic aneurysm; CAD, coronary artery disease; CHF, congestive heart failure; CKD, chronic kidney disease; COPD, chronic obstructive pulmonary disease; DM, diabetes mellitus; IQR, inter-quartile range; LOS, length of stay; SD, standard deviation.

### Reasons for Failed a-EVAR

Ambulatory endovascular aneurysm repair failure to discharge occurred in 16 patients. Procedure-related (n=10) factors were conversion to aorto-uni-iliac endograft (n=3), leg ischemia (n=2), groin hematoma (n=2), inadvertent renal artery coverage (n=1), urinary catheter-related injury (n=1), and endoleak on completion angiogram (n=1). All three patients converted to aorto-uni-iliac repair had failure of contralateral gate cannulation on the original bifurcated endograft. The patients with leg ischemia and groin hematoma were as a result of percutaneous access complications; both patients with leg ischemia required femoral exploration and primary repair. Non-procedure-related factors (n=5) were patients living far (n=3) and old age (n=2). No reason was identified for one patient.

### Thirty-Day and 1-Year EVAR Complication Rates

The 30-day and 1-year rates of ED visits, readmission, and reinterventions were similar between the two cohorts (Supplemental Table 1). At 1 year, there was a non-significant increase in reinterventions (17% a-EVAR and 9% i-EVAR).

### Cost Differences at 30 Days and 1 Year

Cost details are shown in Tables 2 and 3, and Supplemental Figures 1 and 2. Operative costs were similar both at 30 days and 1 year. There was a reduction in the costs of laboratory tests by 62% in the a-EVAR cohort at 30 days. There was also a trend toward reduced costs of ward stay, pharmacy, and ED visits at 30 days. Total perioperative costs were 27% less expensive (12 326 vs 16 843), but the finding was not statistically significant. There was a similar non-statistically significant reduction by 21% at 1 year (13 907 vs 17 641).

**Table 2.** Cost Breakdown at 30 Days.

	a-EVAR (n=100)	i-EVAR (n=70)	p value
Operative	11 816 (3314)	12 978 (4517)	0.312
Bed occupancy			
PACU	542 (499)	549 (372)	0.921
Ward stay	236 (896)	1808 (7294)	0.077
Supportive services			
Blood bank	64 (260)	259 (1483)	0.280
Imaging	189 (218)	299 (530)	0.103
Intervention	620 (3424)	336 (412)	0.491
Labs	35 (102)	92 (212)	0.038
Pharmacy	135 (264)	726 (2828)	0.086
Ancillary care	225 (363)	614 (2398)	0.183
Follow up in clinic	36 (75)	101 (400)	0.183
ED	106 (461)	136 (540)	0.081
Total	12 326 (7110)	16 843 (22 290)	0.105

Abbreviations: ED, emergency department; PACU, post-anesthesia care unit.

**Table 3.** Cost Breakdown at 1 Year.

	a-EVAR (n=100)	i-EVAR (n=70)	p value
Operative	12 587 (5623)	12 978 (4517)	0.793
Bed occupancy			
PACU	561 (505)	549 (372)	0.862
Ward stay	591 (2021)	2050 (8761)	0.175
Supportive services			
Blood bank	107 (460)	260 (1482)	0.332
Imaging	387 (482)	403 (616)	0.850
Intervention	686 (3438)	360 (470)	0.433
Labs	69 (160)	123 (265)	0.095
Pharmacy	205 (416)	771 (2957)	0.116
Ancillary care	337 (621)	721 (2928)	0.283
Follow-up in clinic	112 (168)	170 (500)	0.351
ED	269 (896)	192 (582)	0.533
Total	13 907 (8864)	17 641 (25 067)	0.171

Abbreviations: ED, emergency department; PACU, post-anesthesia care unit.

### Factors Associated With Increased EVAR Costs

A multiple regression model was run to predict factors associated with increased costs at 1 year. Variables included were age, sex, AAA diameter, 30-day reintervention, 30-day readmission, 30-day ED visits, and failed a-EVAR. The model predicted 1-year costs, *F*(7,85)=6.931, p<0.001, *R*^2^=0.311. Age>85 years (*t*=2.343, p=0.021), female sex (*t*=2.175, p=0.032), and 30-day ED visits (*t*=2.808, p=0.006) predicted increased costs. Failed a-EVAR did not predict increased costs (*t*=0.963, p=0.338).

### A-EVAR Versus i-EVAR Costs

**Figure 1.**
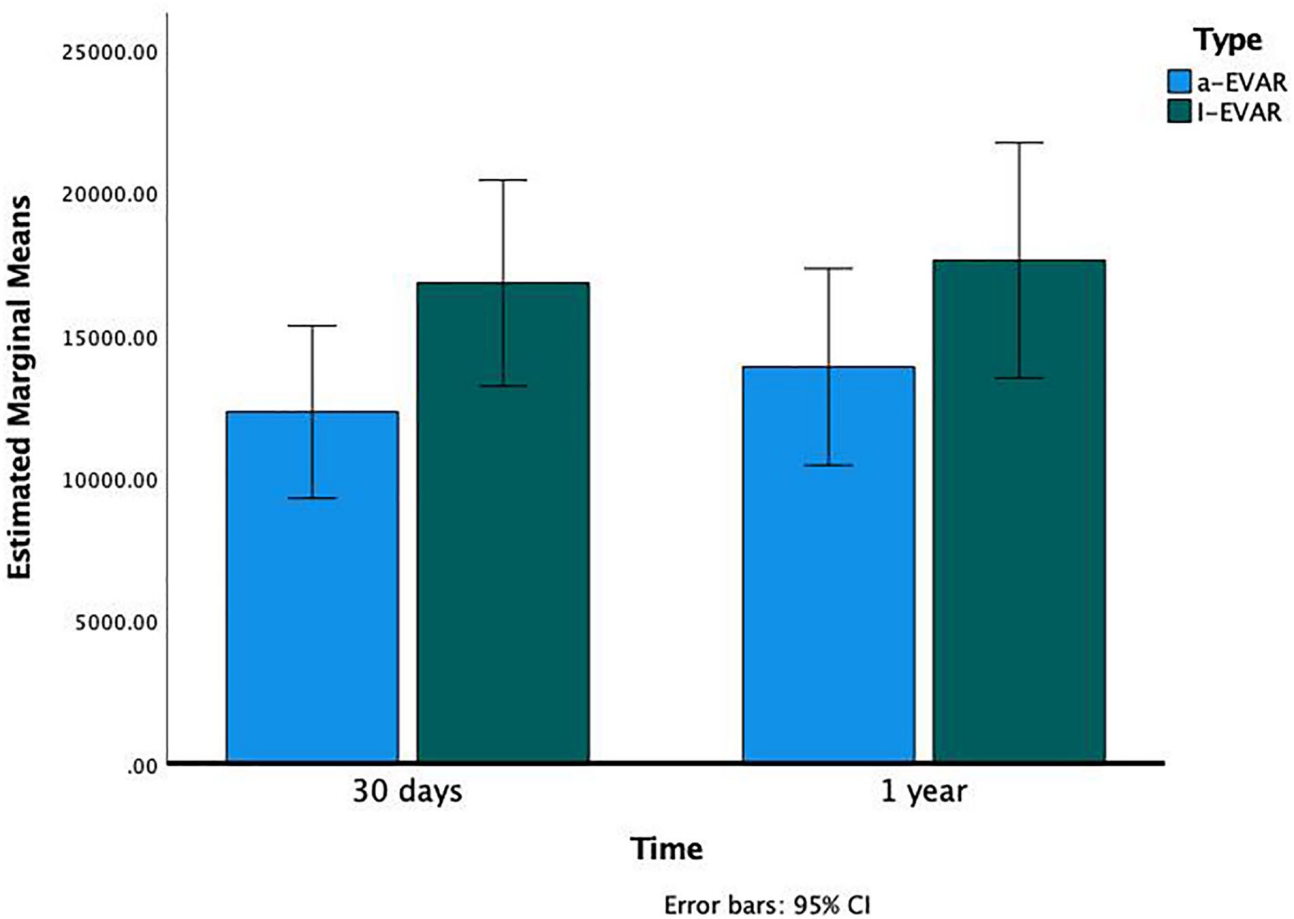
Comparison of 30-day and 1-year costs in a-EVAR and i-EVAR patients. Abbreviations: a-EVAR, ambulatory endovascular aneurysm repair; i-EVAR, inpatient endovascular aneurysm repair.

**Figure 2.**
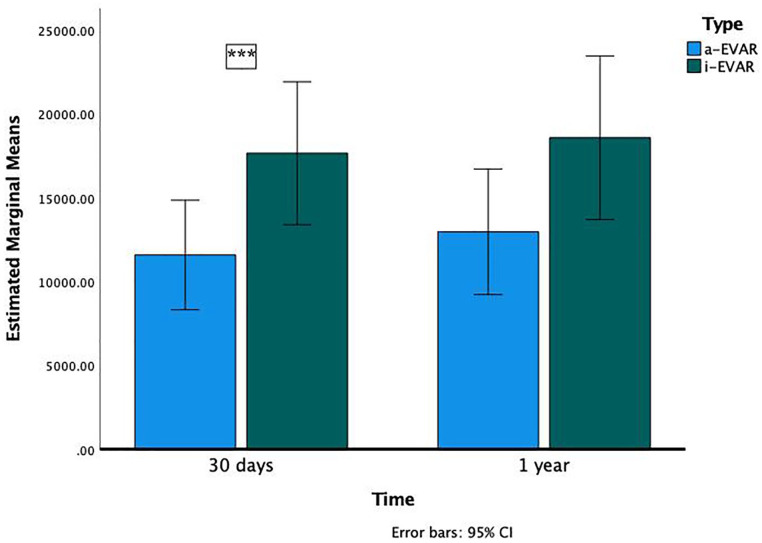
Comparison of 30-day and 1-year costs in a-EVAR and i-EVAR patients younger than 85 years (asterisks indicate difference statistically significant). Abbreviations: a-EVAR, ambulatory endovascular aneurysm repair; i-EVAR, inpatient endovascular aneurysm repair.

**Figure 3.**
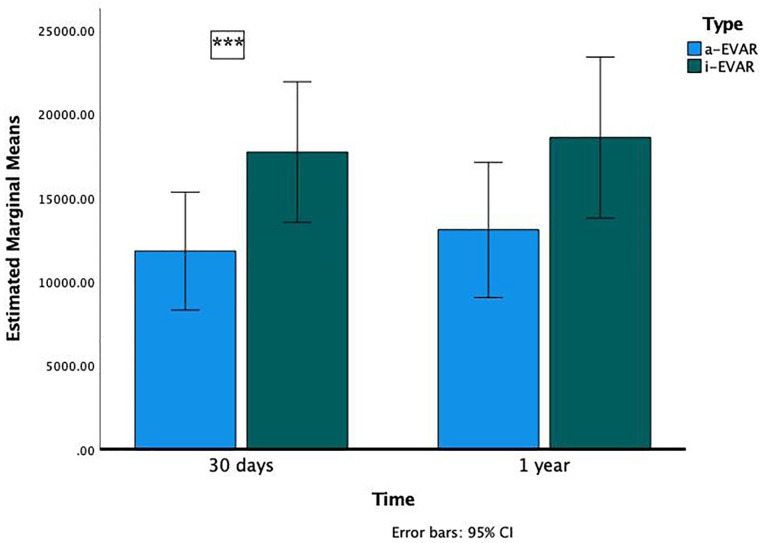
Comparison of 30-day and 1-year costs in a-EVAR and i-EVAR male patients (asterisks indicate difference statistically significant). Abbreviations: a-EVAR, ambulatory endovascular aneurysm repair; i-EVAR, inpatient endovascular aneurysm repair.

Repeated measures ANOVA showed a non-statistically significant reduction in EVAR costs (Figure 1) with a-EVAR at 30 days (mean difference –4517, p=0.060) and 1 year (mean difference –3734, p=0.171). When the analysis is performed considering sex and age, a-EVAR patients who were younger than 85 years had a 34% reduction in EVAR costs at 30 days (mean difference –6072, p=0.027) but not at 1 year (mean difference –5612, p=0.073). Male sex also showed a similar 33% cost reduction at 30 days (mean difference –5905, p=0.035) but not at 1 year (mean difference –5.502, p=0.085). These findings are shown in Figures 2 and 3.

## Discussion

This study describes factors associated with cost reduction in patients undergoing a-EVAR in a system with established same-day discharge clinical pathways. These pathways facilitate discharge after a short post-anesthesia care unit (PACU) stay and can reduce bed occupancy costs by up to 35%.^17,18^ We noted that a-EVAR patients had smaller mean AAA size compared with i-EVAR. Despite AAA diameter not being an inclusion criteria, this likely reflects perceived higher complexity of EVAR with larger aneurysms and longer operative time, as previously reported in a large observational study by Columbo et al.^17^ These a-EVAR patients had a shorter median LOS of 0.5 days compared with 1.3 days in i-EVAR, reflecting modern reported EVAR LOS in literature.^19^ In this study, percutaneous access was utilized in the majority of patients and is likely one of the factors responsible for this observed short LOS.^20^

In terms of costs, a-EVAR costs were similar to i-EVAR at 30 days and 1 year. Cost breakdown identified operative costs as the main drivers of cost for both groups. These findings are similar to previous literature.^15,21^ Supportive non-operative costs have been previously observed to cost less in patients discharged on the same day as well.^22^ This observation was reproduced in our study with laboratory, pharmacy, and post-operative ward stay being less expensive with a-EVAR. This likely reflects more refined standardized post-operative pathways. We noted that the benefit of a-EVAR in reducing costs was only evident in patients younger than 85 years and males. The effects of age and sex as predictors of increased costs have not been previously studied. We postulate that lack of cost reductions in females is related to a higher incidence of procedure-related complications.^23,24^ Poor female representation in studies has been recognized as a limitation in EVAR literature.^9^

a-EVAR success was 84% in our cohort. Lachat et al^25^ and Hanley et al^16^ reported a 92% and 85% same-day discharge success rate, respectively, in patients undergoing EVAR. The profile of patients included in these two studies were very similar to ours with exclusion of patients with complex AAA anatomy, severe comorbidities and patients with poor access to the ED in case of post-operative complications.^16,25^ In terms of costs, planned but failed a-EVAR did not predict increased EVAR costs compared with planned i-EVAR. This could be explained by the fact that majority of the reasons for failure in our study, such as conversion to aorto-uni-iliac repair, access hematoma, traumatic urinary catheterization, and the fact that the patient lives far do not require prolonged treatment or intervention; rather just a transition to i-EVAR. Montross et al^26^ noted a similar finding where only adverse events which required treatment longer than 6 hours predicted against candidacy for same-day discharge.

A review of short stay EVAR by Shaw et al^27^ concluded that it is safe in the low risk patient and results in significant cost reduction. Our study adds to the existing literature as it reports a large institutional experience with a-EVAR. It consolidates the notion that same-day discharge is safe in selected patients.^16,18,22,26,28^ It also identifies up to one-third cost benefit in patients who are younger than 85 years and males in the short-term.

## Conclusion

Same-day discharge is feasible and successful in selected patients. Same-day discharge EVAR shows a trend toward reduced total costs in the short-term, specifically in non-operative costs. Patients younger than 85 years and males show significant short-term cost benefit with EVAR done in the ambulatory setting without increased complications or reinterventions.

## Supplemental Material

sj-docx-1-jet-10.1177_15266028221133694 – Supplemental material for Feasibility and Cost Analysis of Ambulatory Endovascular Aneurysm Repair Supplemental material, sj-docx-1-jet-10.1177_15266028221133694 for Feasibility and Cost Analysis of Ambulatory Endovascular Aneurysm Repair by Ahmed A. Naiem, R.J. Doonan, Andre Guigui, Daniel I. Obrand, Jason P. Bayne, Kent S. MacKenzie, Oren K. Steinmetz, Elie Girsowicz and Heather L. Gill in Journal of Endovascular Therapy

sj-jpg-2-jet-10.1177_15266028221133694 – Supplemental material for Feasibility and Cost Analysis of Ambulatory Endovascular Aneurysm Repair Supplemental material, sj-jpg-2-jet-10.1177_15266028221133694 for Feasibility and Cost Analysis of Ambulatory Endovascular Aneurysm Repair by Ahmed A. Naiem, R.J. Doonan, Andre Guigui, Daniel I. Obrand, Jason P. Bayne, Kent S. MacKenzie, Oren K. Steinmetz, Elie Girsowicz and Heather L. Gill in Journal of Endovascular Therapy

sj-jpg-3-jet-10.1177_15266028221133694 – Supplemental material for Feasibility and Cost Analysis of Ambulatory Endovascular Aneurysm Repair Supplemental material, sj-jpg-3-jet-10.1177_15266028221133694 for Feasibility and Cost Analysis of Ambulatory Endovascular Aneurysm Repair by Ahmed A. Naiem, R.J. Doonan, Andre Guigui, Daniel I. Obrand, Jason P. Bayne, Kent S. MacKenzie, Oren K. Steinmetz, Elie Girsowicz and Heather L. Gill in Journal of Endovascular Therapy
