# Supplementary material for: Feasibility and Cost Analysis of Ambulatory Endovascular Aneurysm Repair
Source: J Endovasc Ther. 2022 Nov 8;31(4):576–83. doi: 10.1177/15266028221133694 (PMC11290021; doi:10.1177/15266028221133694)
Supplement: sj-docx-1-jet-10.1177_15266028221133694 – Supplemental material for Feasibility and Cost Analysis of Ambulatory Endovascular Aneurysm Repair [file sj-docx-1-jet-10.1177_15266028221133694.docx]

|  | **a-EVAR (n=100)** | **i-EVAR (n=70)** | **p-value** |
| --- | --- | --- | --- |
| 30 days, n (%) |  |  |  |
| ED visit | 20 (20%) | 9 (13%) | .223 |
| Readmission | 6 (6%) | 6 (9%) | .519 |
| Reintervention | 7 (7%) | 3 (4%) | .459 |
| 1 year, n (%) |  |  |  |
| ED visit | 31 (31%) | 16 (23%) | .243 |
| Readmission | 14 (14%) | 8 (11%) | .623 |
| Reintervention | 17 (17%) | 6 (9%) | .114 |

**Supplemental Table I - Details of 30 days and 1 year ED visits, reintervention and readmission rates**

ED= Emergency department
