# Supplementary figures and images for: Feasibility and Cost Analysis of Ambulatory Endovascular Aneurysm Repair
Source: J Endovasc Ther. 2022 Nov 8;31(4):576–83. doi: 10.1177/15266028221133694 (PMC11290021; doi:10.1177/15266028221133694)

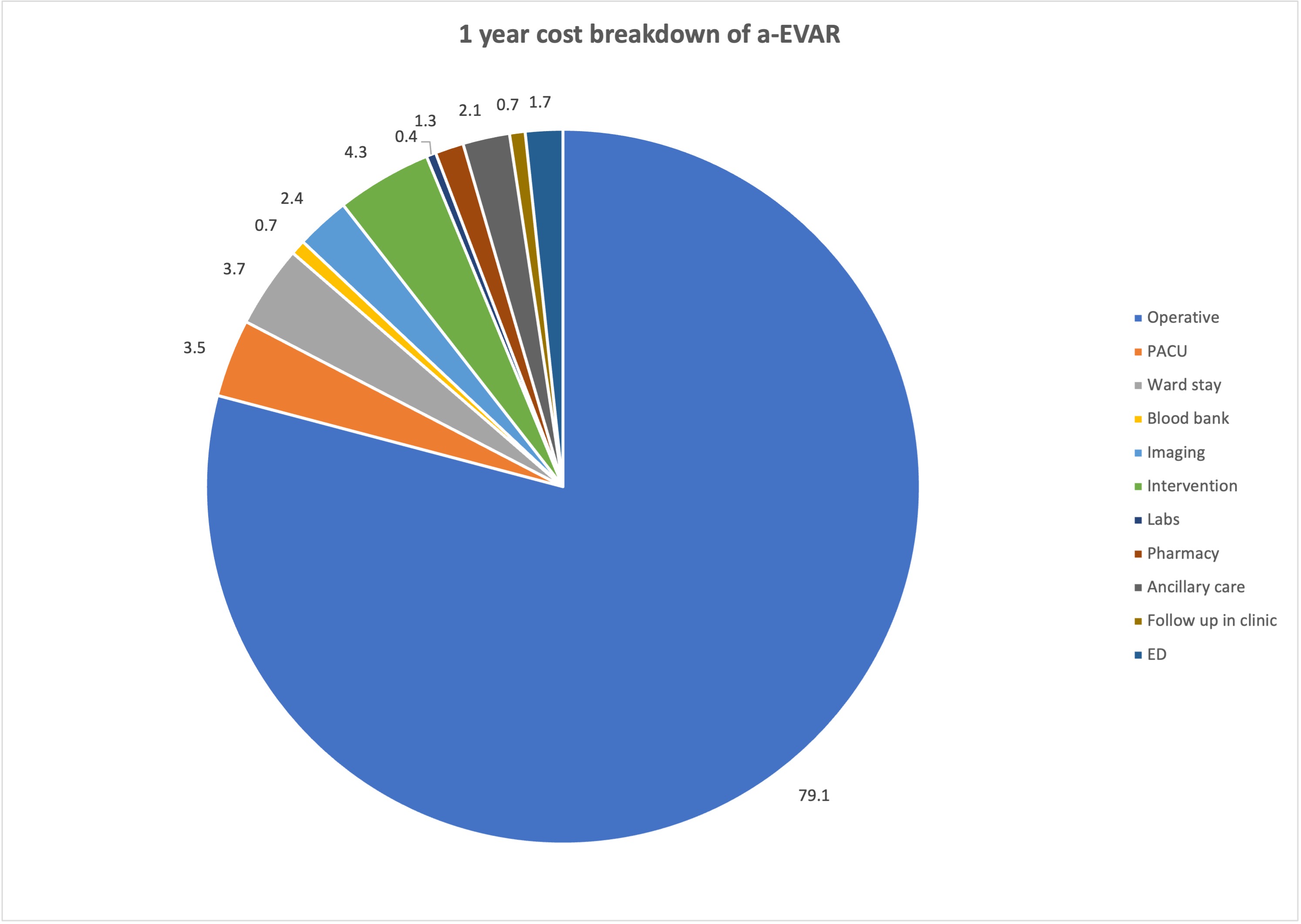

Supplement: sj-jpg-2-jet-10.1177_15266028221133694 – Supplemental material for Feasibility and Cost Analysis of Ambulatory Endovascular Aneurysm Repair [file sj-jpg-2-jet-10.1177_15266028221133694.jpg]

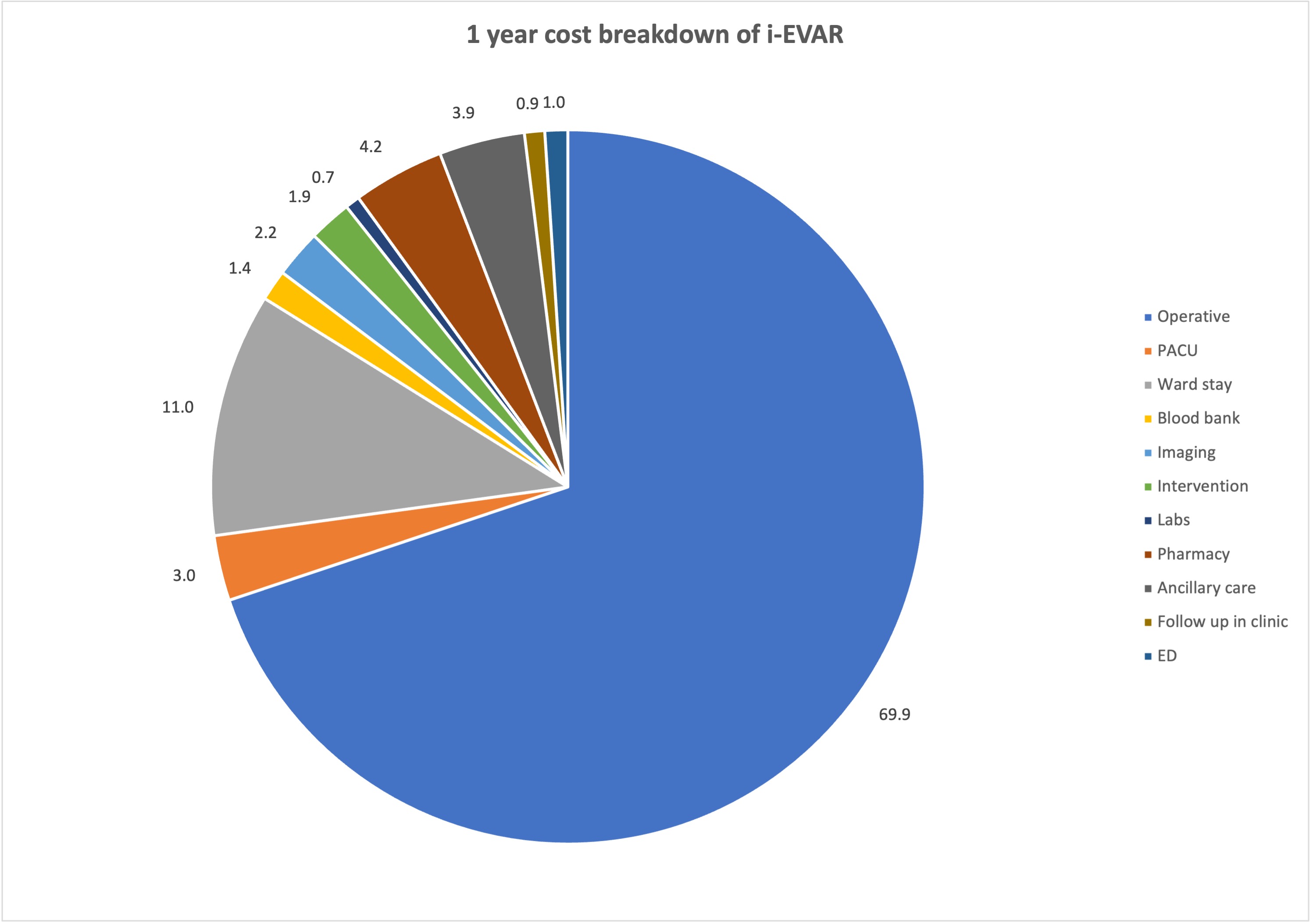

Supplement: sj-jpg-3-jet-10.1177_15266028221133694 – Supplemental material for Feasibility and Cost Analysis of Ambulatory Endovascular Aneurysm Repair [file sj-jpg-3-jet-10.1177_15266028221133694.jpg]
